# From Recommendation to Reality: Implementing Global Lung Initiative (GLI) Global Amid Uncertainty

**DOI:** 10.1016/j.chpulm.2026.100267

**Published:** 2026-05-22

**Authors:** Darshali A. Vyas, Sean D. Levy

**Affiliations:** a Richard A. and Susan F. Smith Center for Outcomes Research, Beth Israel Deaconess Medical Center, Boston, MA; b Division of Pulmonary and Critical Care Medicine, Massachusetts General Hospital, Boston, MA; c Division of Pulmonary, Critical Care Medicine, & Sleep Medicine, Beth Israel Deaconess Medical Center, Boston, MA; d Harvard Medical School, Boston, MA

Reflecting the growing recognition that race is not a biological risk factor for disease, the last 5 years have seen accelerated progress in ending the use of race correction in clinical algorithms.^1^ At least 8 algorithms have been revised without race adjustment since 2020, ushering in a new implementation phase of the movement focused on introducing newly updated algorithms into clinical settings.^2^ Among these changes, race-neutral Global Lung Initiative (GLI) Global equations for interpreting pulmonary function tests (PFTs)^3^ pose unique implementation challenges. Unlike web-based risk scores like the new PREVENT calculator or revised Vaginal Birth After Cesarean (VBAC) calculator that lend themselves to immediate online updates,^2^ changes to PFT interpretation fundamentally alter how we evaluate organ function and understand patients’ lung health. These changes require wider scale investment to expand and modernize laboratory capabilities and commitments from health systems before professional society consensus can translate to widespread clinical change.

Unlike other changes to organ function testing such as the race-free estimated glomerular filtration rate for evaluating renal function, there is no gold standard metric for pulmonary function to adjudicate which equations are correct, creating uncertainty about the transition to GLI Global that has slowed uptake when compared with other race-neutral updates. Despite official recommendations from the American Thoracic Society (ATS) to end race adjustment in PFTs, many health systems have not yet transitioned to GLI Global and some have even rejected the recommendation. As an example, the US Department of Veterans Affairs (VA) suspended their transition to GLI Global in response to 2 studies published soon after the ATS recommendations: (1) Diao et al,^4^ using modeling data, forecasted redistribution of disability benefits to Black veterans; and (2) Bonner et al,^5^ who published data suggesting that surgeons may be deterred from offering lung cancer resection to Black patients if their spirometry values are recategorized as being lower relative to others of the same age, height, and sex at birth. The VA, despite quickly adopting race-neutral estimated glomerular filtration rate reporting in 2021, paused the transition to GLI Global citing the need for additional evidence regarding these 2 concerns.^6^^,^^7^ Although the VA’s decision has been publicly reported, the degree of uncertainty and rates of implementation of GLI Global equations across the field at large remain poorly uncharacterized.

In the previous issue of *CHEST Pulmonary*, Brems et al^8^ begin to fill this gap by assessing the uptake of GLI Global and characterizing provider attitudes surrounding the transition. Among a sample of 66 providers, approximately one-half reported that their PFT laboratories have adopted the new race-neutral GLI Global equations. The study is limited by a low response rate (29%) and an overall small and homogenous sample of academic clinicians who were approached to participate through membership in either the American Lung Association or the Pulmonary Fibrosis Foundation. Because the sample was enriched for academic settings that are likely to have higher rates of exposure to and alignment with recent guideline changes, these findings suggest the national adoption rate (including nonacademic settings) may be even lower than reported in this study.

Among those surveyed, 64% of clinicians agreed with the ATS recommendation to adopt the race-neutral GLI Global. When asked to describe possible disadvantages of using GLI Global, respondents expressed uncertainty over the precision of new spirometry values and the resulting accuracy of disease characterization; notably, no respondents described concerns over redistribution of disability benefits or shifting candidacy for lung cancer resection surgery. Although these 2 concerns have risen to the forefront of the academic debate, this study suggests that practicing pulmonologists may be more concerned with whether their patients are being correctly categorized as having either normal lung function or true lung disease. Importantly, early evidence is reassuring on this front: not only does GLI Global increase detection of lung disease among Black patients, but those recategorized as having abnormal lung function also exhibit accelerated lung function decline over time, suggesting GLI Global is correctly identifying true lung pathology that would be otherwise overlooked with race-specific PFTs.^9^

Of note, although most surveyed clinicians supported the change to GLI Global and cited equity as the major motivation for their support, only one-half could correctly answer 2 content-based questions around how spirometry values would change with the new equations. This discordance suggests that ideologic alignment with the change to GLI Global may not necessarily equate to full mechanistic understanding. This is an important signal that further education is critical, even among centers that have already transitioned to GLI Global and for practitioners in support of the change. Without fully appreciating the nuances of the changes, clinicians will struggle to anticipate the downstream consequences of these updates, advocate for patients when necessary, and explain the changes to their patients. For instance, although GLI Global averages prior race-specific coefficients for spirometry interpretation, these changes do not extend to lung volumes or Diffusion capacity of the lung for carbon monoxide (Dlco), which still use data derived solely from European populations.^3^ Providers should remain aware of this inconsistency as the field moves toward a more unified approach to pulmonary function testing interpretation.

Like many complex management decisions in pulmonary medicine, the implementation of race-neutral GLI Global challenges the field to act even in the face of uncertainty and incomplete data. Awaiting further evidence may be tempting, but this strategy sidesteps a crucial point: although there are some unknowns around the consequences of introducing GLI Global, there is clear evidence of harm from the continued use of race-specific coefficients. Several studies have now documented tangible delays in detection, diagnosis, and management of lung disease among Black patients resulting from race-corrected formulas.^10^, ^11^, ^12^ In the face of this signal toward harm and professional society consensus, health systems should take steps to adopt the newest guideline-concordant care for measuring lung function by transitioning to GLI Global.

However, the implementation process for GLI Global does not end with adoption of the formulas. The transition will require ongoing monitoring and education efforts across disciplines to ensure alignment and coordination of care. Although downstream consequences, such as shifts in lung cancer resection rates, warrant careful study, they should not be taken as a referendum on the appropriateness of GLI Global, but instead as a measure of how effectively its implementation has been communicated to and adopted by affected subspecialties. Changes in receipt of disability benefits should also be reframed as an overdue recalibration instead of a redistribution as we realign our measures with the most updated understandings of race, biology, and disease. Finally, as this study highlights, clinicians are ultimately most concerned with whether they are doing right by their patients. Education on the existing evidence base for GLI Global, rationale for the change, and potential downstream consequences will be critical in achieving greater uptake and confidence in race-neutral PFT interpretation during this next implementation phase. Rather than halting progress in the face of uncertainty, the challenge is to move forward thoughtfully—with a clear-eyed focus on protecting the most vulnerable patients, ensuring that our metrics remain scientifically rigorous, and advancing equity.

## Funding/Support

This study was funded by the 10.13039/100000050National Heart, Lung, and Blood Institute [Grant T32HL116275 to D. A. V.).

## Financial/Nonfinancial Disclosures

The authors have reported to *CHEST Pulmonary* the following: D. A. V. and S. D. L. are both members of the American Thoracic Society Pulmonary Function Test Equity Alliance.
